# Herpes zoster associated with stroke incidence in people living with human immunodeficiency virus: a nested case–control study

**DOI:** 10.1186/s12879-023-08628-8

**Published:** 2023-09-28

**Authors:** Han-Chang Ku, Yi-Lin Wu, Hei-Tung Yip, Cheng-Yang Hsieh, Chung-Yi Li, Huang-Tz Ou, Yen-Chin Chen, Nai-Ying Ko

**Affiliations:** 1 Department of Nursing, Chang Gung University of Science and Technology, Chiayi Branch, Chiayi, Taiwan; 2 Department of Nursing, College of Medicine, National Cheng Kung University Hospital, National Cheng Kung University, Tainan, Taiwan; 3 Clinical Trial Research Center (CTC), China Medical University Hospital, Taichung, Taiwan; 4 Institute of Clinical Pharmacy and Pharmaceutical Sciences, College of Medicine, National Cheng Kung University, Tainan, Taiwan; 5 Department of Neurology, Tainan Sin Lau Hospital, Tainan, Taiwan; 6 Department of Public Health, College of Medicine, National Cheng Kung University, Tainan, Taiwan; 7 Department of Public Health, College of Public Health, China Medical University, Taichung, Taiwan; 8 Department of Healthcare Administration, College of Medical and Health Science, Asia University, Taichung, Taiwan; 9 Department of Pharmacy, National Cheng Kung University Hospital, Tainan, Taiwan; 10 Department of Nursing, College of Medicine, National Cheng Kung University, 1 University Road, Tainan, 7010 Taiwan

**Keywords:** Nested case–control study, Stroke, Incidence, HIV, Herpes zoster, Taiwan National Health Insurance Research Database

## Abstract

**Background:**

The incidence of stroke is increasing among younger people with human immunodeficiency virus (HIV). The burden of stroke has shifted toward the young people living with HIV, particularly in low- and middle-income countries. People infected with herpes zoster (HZ) were more likely to suffer stroke than the general population. However, the association of HZ infection with the incidence of stroke among patients with HIV remains unclear.

**Methods:**

A nested case–control study was conducted with patients with HIV registered in the Taiwan National Health Insurance Research Database in 2000–2017. A total of 509 stroke cases were 1:10 matched to 5090 non-stroke controls on age, sex, and date of first stroke diagnosis. Logistic regression models were used to estimate the odds ratio and 95% confidence intervals (CI) of stroke incidence.

**Results:**

The odds ratio of stroke was significantly higher in the HIV-infected population with HZ (adjusted odds ratio [AOR]: 1.85, 95% CI: 1.42–2.41). A significantly increased AOR of stroke was associated with hypertension (AOR: 3.53, 95% CI: 2.86–4.34), heart disease (AOR: 2.32, 95% CI: 1.54–3.48), chronic kidney disease (AOR: 1.82, 95% CI: 1.16–2.85), hepatitis C virus infection (AOR: 1.49, 95% CI: 1.22–1.83), hyperlipidemia (OR: 1.41, 95% CI: 1.12–1.78), and treatment with protease inhibitors (AOR: 1.33, 95% CI: 1.05–1.69).

**Conclusions:**

Our findings suggest that HZ concurrent with HIV may increase the risk of stroke. The incidence rates of stroke were independent of common risk factors, suggesting strategies for early prevention of HZ infection among people living with HIV.

**Supplementary Information:**

The online version contains supplementary material available at 10.1186/s12879-023-08628-8.

## Introduction

Stroke is the second most common cause of death resulting in a significant health burden worldwide [1]. In 2019, global stroke incidence increased by 70%, its mortality by 43%, and disability-adjusted life-years (DALYs) due to stroke by 32% [2]. An increasing number of individuals are experiencing hemorrhagic stroke with an 80% mortality rate at a younger age in low- and middle-income countries [3, 4].

Given the increased global access to antiretroviral therapy (ART), neurological conditions are becoming more frequent in the aging population with longstanding human immunodeficiency virus (HIV) infection, including cerebrovascular disease (CVD) and peripheral neuropathy [5]. Patients who suffer HIV-induced stroke tend to be younger and more immunocompromised than the general population [6, 7]. People living with HIV (PLWH) in Europe and America have a greater risk of stroke than the general population [8–12]. Chronic inflammation and immune activation, typically observed in elderly people and termed “inflammaging,” can occur in HIV-infected patients who experience a unique type of premature aging, which significantly affects their quality of life [13, 14].

HIV-associated vasculopathy encompasses several pathologic phenotypes of stroke found in PLWH [15–17]. This may be due to chronic systemic inflammation, which occurs despite virological suppression and due to long-term ART toxicity, lifestyle factors, or a combination thereof [18, 19]. Two systematic reviews and meta-analyses reported an increased risk of ischemic stroke post-herpes zoster (HZ) infection in younger individuals and those not prescribed antivirals [20, 21]. Male sex, hypertension, metabolic factors, older age, alcohol/drug abuse, HIV RNA, high viral load, and CD4 count < 200 cells/μL have more strongly predicted ischemic stroke [9, 10, 22, 23]. Conversely, HZ infection, male sex, cardiovascular history, hypertension, smoking, injecting drug users (IDU), hepatitis C, and estimated glomerular filtration (eGFR) rate < 60 mL/min/m^3^ and CD4 count < 200 cells/μL have strongly predicted hemorrhagic stroke [8, 22, 24, 25]. A pilot study in Uganda reported that an HIV-infected population with varicella zoster virus antibodies had a three-fold higher risk of stroke [26].

Herpes zoster (shingles) is caused by the reactivation of the varicella-zoster virus (VZV), which typically remains latent after primary infection with varicella (chickenpox) during childhood. Chickenpox is characterized by vesicular lesions on an erythematous base in different stages of development; lesions are most concentrated on the face and trunk [27]. The presenting clinical manifestations of herpes zoster are usually rash and acute neuritis. HZ (shingles) is caused by the first VZV, which remains latent in the sensory ganglia following varicella infection [28]. HZ is largely considered to be a once-in-a-lifetime experience, up to 20 percent of people will develop shingles during their lifetime. HZ recurrence is thought to be limited to immunocompromised individuals and is an HIV-associated opportunistic infection. Herpes zoster (HZ) infection is a risk factor for stroke. People with HZ infection were found to be more likely to suffer stroke than the general population with the incidence rate ratios (IRRs) for all patients and patients aged 18 to 49 years were 1.40 and 8.12, respectively [29]. An analysis of records from the Taiwan National Health Research Institute revealed a 30% increase in the risk of stroke among the general population within 1 year of having an HZ infection [24]. Furthermore, the relationship between the HZ infection and the risk of stroke among people with HIV infection remains unclear.

This study aimed to estimate the odds ratio of stroke in association with HZ among PLWH. In the present study, we used the linked data obtained from the Taiwan National Health Insurance database to conduct a population-based, nested case–control study. The primary objective was to investigate the potential association between HZ infection and an elevated risk of stroke incidence. Additionally, we sought to contrast the occurrence of stroke events in HIV-infected patients both with and without a history of HZ infection.

## Methods

### Data source

Patient data were retrieved from medical claims of Taiwan’s National Health Insurance (NHI) program. The NHI released de-identified secondary data to the public for research purposes. Access to the NHI medical claims was approved by the Review Committee of Health and Welfare Data Science Center, Ministry of Health and Welfare. Our study was approved by the Research Ethics Committee of Tainan Municipal An-Nan Hospital-China Medical University (approval number TMANH109-REC024). The NHI medical claims cover claims data of > 99% of Taiwan residents [30]. Emergency, outpatient, and inpatient medical claims of all diagnosed HIV-infected persons from 2000 to 2018 were retrieved from the NHI medical claims. Data linkages among various medical claim datasets were allowed using de-identified personal identification numbers.

### Nested case–control design and participants

The case–control study was nested within 35,168 patients with HIV who had ≥ 1 admission or ≥ 2 ambulatory care visits for primary HIV diagnosis (International Classification of Diseases, Ninth Revision, Clinical Modification [ICD-9-CM]) code: 042–044.9, V08; (International Classification of Diseases, Tenth Revision, Clinical Modification [ICD-10-CM]) code: B20-B24, Z21, Z22.6) within a 365-day period in 2002–2016. The following HIV patients were excluded: Patients with HIV aged < 18 years old or unknown sex (*n* = 699). In measuring the 2-year period of case management follow-up, patients diagnosed with HIV in 2000 and 2001 were excluded (*n* = 2,769). A final total of 31,707 patients with HIV was included in the study population.

### Selection of cases and controls

We estimated the incidence of stroke in HIV-infected patients who had their first emergency visit or hospitalization for stroke events in the period from the date of the initial diagnosis (i.e., index date) to the end of the follow-up period, date of death, or December 31, 2017. By modifying the coding algorithm proposed by Hsieh et al., [31] the incidence of major stroke events is defined as the first occurrence of strokes in this study, including ischemic stroke (ICD-9-CM: 433.xx, 434.xx, and ICD-10-CM: I63), hemorrhagic stroke (ICD-9-CM: 430, 431, and ICD-10-CM: I60, I61) and transient ischemic attack (TIA) (ICD-9-CM: 435.xx, and ICD-10-CM: I60-64, G45) in emergency or inpatient medical records from the first clinical visit for HIV to the end of the observation period [32, 33]. A total of 509 patients aged ≥ 18 years and with an incident stroke were identified. In our study, the diagnosis of HIV followed by the occurrence of stroke indeed constitutes a rare event. We have opted for a larger matching ratio of 1:10. This choice aligns with our goal of enhancing comparability and minimizing potential biases between the two groups. For patients with stroke, ten controls were randomly selected using propensity score matching to identify the stroke (*n* = 590) and non-stroke (*n* = 5,090) groups by matching stroke cases on sex, age (± 5 years), and month of stroke diagnosis. After propensity score matching, including the month of diagnosis, age (± 5 years) at diagnosis, and sex, were comparable between the stroke and non-stroke groups.

The date of the first-ever stroke diagnosis in 2002–2017 was considered as the index date for each patient with stroke and his/her matched controls. Therefore, an individual who was selected as a control could later become a stroke case. In all, 5,090 matched control subjects were selected.

**Fig. 1:**
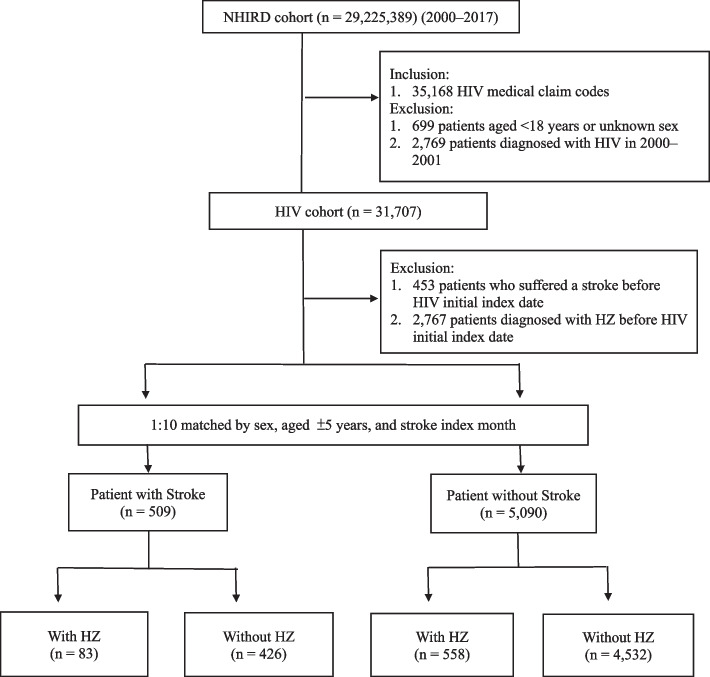
Flow chart of patient selection for the case and control groups

The HZ group included all patients who had ≥ 1 admission or ≥ 2 ambulatory care visits before the first stroke event after HZ diagnosis (ICD-9-CM: 053.0–053.11, 053.19–053.9, and ICD-10-CM: B02.xx) events were identified based on the medical claims of inpatient or outpatient department. These patients were required to have at least two outpatients (within 7 days apart) or inpatient visits for HZ after the initial HIV diagnosis. The incidence of HZ was defined as 1 month after an HIV diagnosis because most of the people living with HIV infection had a concurrent HZ infection at the time of diagnosis. Based on the incidence of HZ within the 1-year period between the enrollment and index dates, we further categorized the study subjects into four groups, namely, stroke patients with and without HZ and controls with and without HZ. A flow chart of the patient enrollment process is illustrated in Fig. 1.

### Confounders

Demographic and risk factors were also retrieved from the claims data, including age at the date of the first HIV and stroke diagnoses, geographic area, and monthly income after the HIV diagnosis. Patients’ demographic characteristics, including age, sex, income, and residential area, were retrieved from beneficiary records. The urbanization level for each 316 city/township in Taiwan was determined using Liu et al.’s method which classified all cities and townships in Taiwan into different urbanization levels based on various indicators [34]. The median family annual income of each city/township was used to indicate the neighborhood socioeconomic of status for each study patient. Monthly income-based insurance premium was categorized into three levels with cut-off points of < 20,000 New Taiwan dollars (NTD) (< 660.50 USD), 20,000–40,000 NTD (660.50–1321.00 USD), and > 40,001 NTD (> 1321.04 USD) (1 USD = 30.28 NTD, Exchange Rate for Individual Income Tax Return from Year 2015 to Year 2022).

Various comorbidities were considered as risk factors for stroke: hypertension, diabetes mellitus, hyperlipidemia, CVD, coronary artery disease, myocardial infarction, heart failure, atrial fibrillation, chronic kidney disease, hepatitis B virus (HBV), and hepatitis C virus (HCV) (Supplemental Table 1). Each comorbidity was identified following the relevant ICD-9-CM or ICD-10-CM codes presented in three outpatient claims within 1 year before the index date.

Since 2015, the World Health Organization has recommended that PLWH undergo ART, irrespective of CD4 count or clinical stage. The ART regimen groups were classified into three groups: non-nucleoside reverse transcriptase inhibitors (NNRTIs), nucleoside reverse transcriptase inhibitors (NRTIs), and protease inhibitors (PIs). To mitigate potential confounding effects, we diligently excluded patients concurrently receiving both NNRTIs and PIs regimens during the drug selection process. These groups were based on stable users (defined as continuous use for up to ≥ 6 months) after the ART initiation. Patients who received ART more than 6 months before the index date of stroke occurrence were defined as ART users. Baseline antiviral medications for HZ were recorded as acyclovir, valacyclovir, and famciclovir administered for 7–10 days. We retrieved information on all acyclovir (ATC code: J05AB01), valacyclovir (ATC code: J05AB11), and famciclovir (ATC code: J05AB09) prescriptions.

### Statistical analysis

First, the difference between the characteristics of the stroke and control groups was assessed using the chi-squared test. To examine the correlation between the risk of stroke in people living with HZ, the crude odds ratios (ORs), covariate-adjusted odds ratios (AORs), and 95% confidence intervals (CIs) were calculated from conditional logistic regression analysis. The regression model was created to investigate whether exposure to HZ may pose a duration effect on the risk of stroke incidence. The ORs and AORs were also used to estimate the relative stroke risk at various time points after determining the duration of HIV infection and concurrent HIV and HZ infection. The potential confounders adjusted for in the multivariate regression model included all variables listed in Table 1. All statistical analyses were performed using the SAS version 9.4 software (SAS Institute, Cary, NC, USA). A *p*-value of < 0.05 was considered statistically significant.

**Table 1:** Characteristics of patients and controls

Variable	Cases (stroke) (*n* = 509) n (%)	Controls (non-stroke) (*n* = 5,090) n (%)	*p*-value
Age (years)			0.98
18–34	80 (15.72)	805 (15.81)	
35–44	159 (31.24)	1591 (31.26)	
45–54	160 (31.43)	1644 (32.30)	
55–64	84 (16.50)	787 (15.46)	
≥ 65	26 (5.11)	263 (5.17)	
Mean (SD)	46.14 (11.17)	45.73 (11.11)	
Sex			0.53
Female	46 (9.04)	419 (8.23)	
Male	463 (90.96)	4671 (91.77)	
Geographic area			** < 0.001**
North	236 (46.37)	2773 (54.48)	
Central	112 (22.00)	985 (19.35)	
South	158 (31.04)	1248 (24.52)	
East	3 (0.59)	84 (1.65)	
Monthly income, USD			** < 0.001**
< 660.50	333 (65.42)	2368 (46.52)	
660.50–1321.00	148 (29.08)	1840 (36.15)	
> 1321.04	28 (5.50)	882 ((17.33)	
Comorbidity			
Hypertension	273 (53.63)	1338 (26.29)	** < 0.001**
HCV	230 (45.19)	1597 (31.38)	** < 0.001**
Hyperlipidemia	152 (29.86)	1191 (23.40)	**0.001**
HBV	100 (19.65)	897 (17.62)	0.26
Heart disease	41 (8.06)	122 (2.40)	** < 0.001**
Diabetes mellitus	40 (7.86)	292 (5.74)	0.053
Chronic kidney disease	33 (6.48)	133 (2.61)	** < 0.001**
Coronary artery disease	15 (2.95)	94 (1.85)	0.087
Alcohol-related illness	15 (2.95)	78 (1.53)	0.017
PAOD	5 (0.98)	14 (0.28)	**0.009**
AMI	0 (0)	16 (0.31)	**-**
ART regimen			
PIs	116 (22.79)	889 (17.47)	**0.003**
NRTIs	88 (17.29)	878 (17.25)	0.98
NNRTIs	17 (3.73)	123 (2.42)	0.20

*SD *Standard deviation, *USD *United States Dollars, *HCV *Hepatitis C virus, *HBV *Hepatitis B virus, *PAOD *Peripheral arterial occlusive disease, *AMI *Acute myocardial infarction, *ART *Antiretroviral therapy, *PIs *Protease inhibitors, *NRTIs *Nucleoside reverse transcriptase inhibitors, *NNRTIs *Non-nucleoside reverse transcriptase inhibitors

## Results

From January 1, 2000, to December 31, 2017, a total of 509 cases diagnosed with HIV infection who had strokes were investigated, whereas 5,090 controls with HIV who had not suffered from a stroke at the index date were considered as controls, showing that study patients with stroke tended to be young, living in urban areas, and earning lesser wages. The mean ages were similar for the patients and controls at 46.14 years and 45.73 years, respectively. Male patients accounted for 90.96% and 91.77% of patients and controls, respectively. The area of residence and monthly income varied significantly among people with HIV infection who suffered from stroke (*p* < 0.0001). Approximately 43.81% of PLWH who suffered a stroke had received ART, with PIs (22.79%) as the primary medications. The age, sex, insurance premium, and income level are comparable in patients and controls in this study. However, compared with controls, patients were more likely to have a history of comorbidities, including alcohol-related illness, hypertension, hyperlipidemia, peripheral arterial occlusive disease, heart disease, chronic kidney disease, and HCV (Table 1).

Table 2 shows the crude ORs, AORs, and 95% CIs for the risk of stroke in PLWH in Taiwan. After controlling the potential confounders including sex, age, geographic area, monthly income, and comorbidity, results of multivariate logistic regression analysis were compared to that of controls, and the people with HIV infection with HZ (AOR: 1.85, 95% CI: 1.42–2.41), hypertension (AOR: 3.53, 95% CI: 2.86–4.34), heart disease (AOR: 2.32, 95% CI: 1.54–3.48), chronic kidney disease (AOR: 1.82, 95% CI: 1.16–2.85), hyperlipidemia (AOR: 1.41, 95% CI: 1.12–1.78), HCV (AOR: 1.49, 95% CI: 1.22–1.83) and taking PIs (AOR: 1.33, 95% CI: 1.05–1.69) have a significantly higher risk of stroke.

**Table 2:** Odds ratios of the risk of stroke in PLWH in Taiwan

Variables	Controls (non-stroke) (*n* = 5,090)	Cases (stroke) (*n* = 509)	Crude OR (95% CI)	*p*-value	Adjusted OR^a^ (95% CI)		*p*-value
HZ							
No	4,532 (89.04%)	426 (83.69%)	Reference		Reference		
Yes	558 (10.96%)	83 (16.31%)	1.58 (1.23–2.03)	** < 0.001**	1.85 (1.41–2.41)	** < 0.001**	
Sex							
Female	419 (8.23%)	46 (9.04%)	Reference		Reference		
Male	4,671 (91.77%)	463 (90.96%)	0.90 (0.66–1.24)	0.53	0.91 (0.65–1.27)	0.59	
Age							
18–34	805 (15.82%)	80 (15.72%)	Reference		Reference		
35–44	1,591 (31.26%)	159 (31.24%)	1.01 (0.76–1.33)	0.96	0.76 (0.56–1.02)	**0.003**	
45–54	1,644 (32.29%)	160 (31.43%)	0.98 (0.74–1.30)	0.72	0.54 (0.39–0.73)	0.45	
55–64	787 (15.46%)	84 (16.50%)	1.07 (0.78–1.48)	0.56	0.44 (0.30–0.63)	**0.02**	
≥ 65	263 (5.17%)	26 (5.11%)	0.99 (0.63–1.58)	0.93	0.35 (0.21–0.58)	**0.007**	
Geographic area							
North	2,773 (54.48%)	236 (46.37%)	Reference		Reference		
Central	985 (19.35%)	112 (22.00%)	1.34 (1.05–1.69)	**0.04**	0.92 (0.81–1.33)	0.24	
South	1,248 (24.52%)	158 (31.04%)	1.49 (1.20–1.84)	**0.007**	1.24 (0.99–1.55)	**0.02**	
East	84 (1.65%)	3 (0.59%)	0.46 (0.13–1.34)	0.06	0.38 (0.12–1.30)	0.1	
Monthly income, USD							
< 660.50	2,368 (46.52%)	333 (65.42%)	Reference		Reference		
660.50–1321.00	1,840 (36.15%)	148 (29.08%)	0.57 (0.47–0.70)	0.16	0.56 (0.45–0.70)	0.3	
> 1321.04	882 (17.33%)	28 (5.50%)	0.23 (0.15–0.33)	** < 0.001**	0.24 (0.16–0.36)	** < 0.001**	
Comorbidity							
Alcohol-related illness							
No	5,012 (98.47%)	494 (97.05%)	Reference		Reference		
Yes	78 (1.53%)	15 (2.95%)	1.95 (1.11–3.42)	**0.02**	1.30 (0.72–2.36)	0.39	
Hypertension							
No	3,752 (73.71%)	236 (46.37%)	Reference		Reference		
Yes	1,338 (26.29%)	273 (53.63%)	3.24 (2.70–3.90)	** < 0.001**	3.53 (2.86–4.34)	** < 0.001**	
Diabetes mellitus							
No	4,798 (94.26%)	469 (92.14%)	Reference		Reference		
Yes	292 (5.74%)	40 (7.86%)	1.40 (0.99–1.98)	**0.05**	0.72 (0.49–1.08)	0.11	
Hyperlipidemia							
No	3,899 (76.60%)	357 (70.14%)	Reference		Reference		
Yes	1,191 (23.40%)	152 (29.86%)	1.39 (1.14–1.70)	**0.001**	1.41 (1.12–1.78)	**0.003**	
PAOD							
No	5,076 (99.72%)	504 (99.02%)	Reference		Reference		
Yes	14 (0.28%)	5 (0.98%)	3.60 (1.29–10.04)	**0.01**	2.61 (0.83–8.17)	0.1	
Coronary artery disease							
No	4,996 (98.15%)	494 (97.05%)	Reference		Reference		
Yes	94 (1.85%)	15 (2.95%)	1.61 (0.93–2.80)	0.09	1.12 (0.60–2.09)	0.72	
Heart disease							
No	5,626 (97.60%)	468 (91.94%)	Reference		Reference		
Yes	122 (2.40%)	41 (8.06%)	3.57 (2.47–5.15)	** < 0.001**	2.32 (1.54–3.48)	** < 0.001**	
Chronic kidney disease							
No	4,957 (97.39%)	476 (93.52%)	Reference		Reference		
Yes	133 (2.61%)	33 (6.48%)	2.58 (1.75–3.83)	** < 0.001**	1.82 (1.16–2.85)	**0.01**	
HBV							
No	4,193 (82.38%)	409 (80.35%)	Reference		Reference		
Yes	897 (17.62%)	100 (19.65%)	1.14 (0.91–1.44)	0.26	1.06 (0.83–1.36)	0.63	
HCV							
No	3,493 (68.62%)	279 (54.81%)	Reference		Reference		
Yes	1,597 (31.38%)	230 (45.19%)	1.80 (1.50–2.17)	** < 0.001**	1.49 (1.22–1.83)	** < 0.001**	
ART regimen							
Only NRTIs							
No	4,212 (82.75%)	421 (82.71%)	Reference		Reference		
Yes	878 (17.25%)	88 (17.29%)	1.00 (0.79–1.28)	0.98	0.99 (0.74–1.31)	0.93	
Only NNRTIs							
No	4,967 (97.97%)	492 (96.66%)	Reference		Reference		
Yes	123 (2.03%)	17 (3.34%)	1.40 (0.83–2.34)	0.2	1.25 (0.68–2.29)	0.47	
Only PIs							
No	4,181 (82.14%)	393 (77.21%)	Reference		Reference		
Yes	889 (17.86%)	116 (22.79)	1.40 (1.12–1.74)	**0.003**	1.33 (1.05–1.69)	**0.02**	

*PLWH* People living with HIV, *OR* Odds ratio, *HZ* Herpes zoster, *USD* United States Dollars, *PAOD* Peripheral arterial occlusive disease, *AMI* Acute myocardial infarction, *HBV* Hepatitis B virus, *HCV* Hepatitis C virus, *ART* Antiretroviral therapy, *NRTIs* Nucleoside reverse transcriptase inhibitors, *NNRTIs* Non-nucleoside reverse transcriptase inhibitors, *PIs* Protease inhibitors

^a^Odds ratios were calculated from the logistic regression model adjusted for age, sex, geographic area, monthly income, comorbidity, and ART regimen

Table 3 shows the crude ORs and AORs for stroke risk based on the duration of concurrent HIV and HZ infection. After controlling the potential confounders, the duration of concurrent HIV and HZ infection at > 3 and ≤ 6 months (AOR: 1.45, 95% CI: 0.71, 2.97), and > 6 months (AOR: 2.04, 95% CI: 1.49, 2.78) showed a higher risk of stroke.

**Table 3:** Odds ratios for stroke risk of the duration of concurrent HIV and HZ infection

Duration of concurrent HZ infection	Non-stroke cases	Stroke cases	OR (95% CI)	*p*-value	Adjusted OR^a^ (95% CI)	*p*-value
≤ 3 months	110	15	1.00 (Reference)		1.00 (Reference)	
> 3 and ≤ 6 months	88	9	1.08 (0.54,2.15)	0.58	1.45 (0.71,2.97)	0.97
> 6 months	360	59	1.73 (1.29,2.31)	**0.02**	2.04 (1.49,2.78)	**0.03**

*HZ* Herpes zoster, *OR* Odds ratio, *CI* Confidence interval

^a^Odds ratios were calculated from the logistic regression model adjusted for age, sex, area of residence, monthly income, comorbidity, and highly active antiretroviral therapy regimen (NNRTIs, NRTIs, PIs)

## Discussion

This is the first population-based study reporting an elevated 1.85-fold risk of stroke in patients with HZ among PLWH. Our study finding is consistent with two cohort studies in America that reported that the stroke incidence was 2.26–5.27 per 1,000 person-years among people living with HIV [9, 35].

Our finding showed that male sex and not receiving ART were the major risk factors for HZ among HIV-infected patients. The majority of stroke occurrence in people living with HIV and HZ patients were men, which differ from the observation made in other studies [5, 9, 36], indicating that stroke among people with HIV infection occurred mostly in women. The majority of people with HIV were men due to the demographics of the HIV-infected population in Taiwan. However, the adjusted hazard ratio of stroke among male and female patients during the 1-year follow-up period was 1.30–1.32 times higher (*p* < 0.05) than that of patients with HZ [24]. In addition, Gutierrez et al. demonstrated that a CD4 count < 200, longer duration of HIV infection, and prior history of stroke were all associated with large-artery atherosclerosis, whereas small-vessel disease was associated with CD4 count > 200, no history of prior cardiac disease, and male sex [37]. Future research is needed to elucidate the pathophysiology of HIV, HZ, and stroke risk and investigate sex differences in stroke risk.

The main age group of people with HIV infection suffering from stroke were in the 35–44 and 45–54 age groups. The results of multivariate logistic regression analysis revealed that people with HIV infection aged 35–44, 55–64, and more than 65 years have a significantly lower risk of stroke, which were inconsistent with those of other studies reporting that a higher incidence of stroke occurs among younger people with HIV infection [9, 16, 36]. Most HZ-associated vasculopathy develop within 6 weeks after HZ events [36]. In the general population, younger people (0–49 years) had a higher risk of developing stroke or TIA within 1 year after HZ diagnosis [38]. Neurological conditions, including stroke, are occurring at a younger age in people with chronic HIV infection, a phenomenon referred to as accelerated aging. This may due to chronic inflammation, which occurs despite virological suppression and due to long-term ART toxicity, lifestyle risk factors, or a combination of the above [5, 19].

Our study has shown a significantly increased risk of stroke following an HIV infection, particularly due to comorbidities such as hypertension, hyperlipidemia, heart disease, chronic kidney disease, HCV, and treatment with PIs. Moreover, the risk of stroke is higher among people with HIV infection with hypertension, a finding consistent with the results of other studies [23, 34, 36, 39]. People with HIV infection have a significantly higher risk of stroke due to comorbidities, such as heart disease and chronic kidney disease, which is consistent with that of the general population. Among the people with HIV infection who underwent ART, particular treatment with PIs, low-density cholesterol, smoking, and high-density cholesterol are associated with heart diseases [23, 36].

Our study indicated that the overall risk of stroke was significantly increased with > 6 months of concurrent HIV and HZ infection. HZ-associated vasculopathy is also defined as follows: (1) induced production of prothrombotic autoimmune antibodies, (2) autoimmune phenomena caused by circulating immune complexes, and (3) disrupted internal elastic lamina, intimal hyperplasia, and decreased smooth muscle cells in the tunica media layer [40, 41]. HIV-associated vasculopathy or histologic evidence of extensive atherosclerosis was observed in relatively younger patients who started ART in the 6 months prior to their stroke event [42]. The rates of stroke were significantly increased within 1 week to 3 months after HZ diagnosis. However, the risk was reduced gradually and resolved after over 1 year [43–45]. Nevertheless, the underlying mechanism of the risk of stroke in concurrent HZ and HIV remains unclear. This should be evaluated in future clinical studies.

This key strength of this study is advantageous because it is a nested case–control study, a research design combining the advantages of case–control and cohort studies. It retains the advantages of the case–control study that saves manpower, material resources, and time, and has the advantage of a cohort study to determine the relationship between exposure and causality. In addition, both the patient and control groups are retracted from the same generational group of patients with HIV, which reduces the sample selection bias. During the process, matched methods are used to randomly select the control group, which improves the statistical efficiency. Few studies have focused on the HZ-induced risk of stroke among people with HIV infection in the ART era.

This study has limitations. First, the secondary health insurance database was analyzed. The potential of missing coding or coding errors may result in misclassifications. Nevertheless, such issues would result in a biased association toward the null effect, thereby underestimating the predictive power of our study. Second, the test data such as viral load and CD4 index and the lack of characterization of stroke pathology were not collected. Therefore, the patient’s immune status, ART compliance with medication, correlation with stroke, and excluding the subset of stroke cases cannot be assessed. Prospective studies can be conducted to assess the relationship between the side effects of using ART and stroke in the future. Furthermore, HIV infection and HZ infection can both induce stroke. We cannot exclude the possibility that there may be a common risk factor for the occurrence of HZ and stroke in the PLWH population. Intermediary analysis can be used to explore the relationship between two diseases and stroke.

## Conclusions

In conclusion, this is the first study in Asia to analyze the risk of stroke in people with HIV infection with HZ from 2000 to 2017. A 1.85-fold increased risk of stroke was observed in patients with HZ among the people with HIV infection. A significantly elevated risk of stroke was found in PLWH with hypertension, heart disease, chronic kidney disease, hyperlipidemia, HCV, and treatment with PIs. Further studies are needed to clarify the relationships between HIV, HZ, and stroke. Clinicians and people with HIV infection should be aware of the risk factors of stroke among PLWH. The results of this study can be used as a basis by which health policymakers can determine appropriate stroke prevention programs for people living with HIV and HZ, as well as to help them formulate and implement regulations related to controlling for risk factors of stroke and stroke prevention among people living with HIV and HZ.

### Supplementary Information

**Additional file 1: Supplementary Table 1.** Operation definitions for study outcomes. **Supplementary Figure 1.** Overview of the participant selection process.

## Data Availability

All data generated or analyzed during this study are included in this published article (and its supplementary information files).

## References

[1] World Health Organization. The top 10 causes of death*.* 2020. https://www.who.int/news-room/fact-sheets/detail/the-top-10-causes-of-death. Accessed 20 May 2022.

[2] Owolabi MO, Thrift AG, Mahal A, Ishida M, Martins S, Johnson WD, Pandian J, Abd-Allah F, Yaria J, Phan HT (2022). Primary stroke prevention worldwide: translating evidence into action. Lancet Public Health.

[3] Katan M, Luft A (2018). Global burden of stroke. Semin Neurol.

[4] Lanas F, Seron P (2021). Facing the stroke burden worldwide. Lancet Glob Health.

[5] Thakur KT, Boubour A, Saylor D, Das M, Bearden DR, Birbeck GL (2019). Global HIV neurology: a comprehensive review. AIDS.

[6] Benjamin LA, Corbett EL, Connor MD, Mzinganjira H, Kampondeni S, Choko A, Hopkins M, Emsley HC, Bryer A, Faragher B (2016). HIV, antiretroviral treatment, hypertension, and stroke in Malawian adults: A case-control study. Neurology.

[7] Breuer J, Pacou M, Gautier A, Brown MM (2014). Herpes zoster as a risk factor for stroke and TIA: a retrospective cohort study in the UK. Neurology.

[8] Chow FC, He W, Bacchetti P, Regan S, Feske SK, Meigs JB, Grinspoon SK, Triant VA (2014). Elevated rates of intracerebral hemorrhage in individuals from a US clinical care HIV cohort. Neurology.

[9] Chow FC, Regan S, Feske S, Meigs JB, Grinspoon SK, Triant VA (2012). Comparison of ischemic stroke incidence in HIV-infected and non-HIV-infected patients in a US health care system. J Acquir Immune Defic Syndr.

[10] Marcus JL, Leyden WA, Chao CR, Chow FC, Horberg MA, Hurley LB, Klein DB, Quesenberry CP, Towner WJ, Silverberg MJ (2014). HIV infection and incidence of ischemic stroke. AIDS.

[11] Rasmussen LD, Engsig FN, Christensen H, Gerstoft J, Kronborg G, Pedersen C, Obel N (2011). Risk of cerebrovascular events in persons with and without HIV: A Danish nationwide population-based cohort study. AIDS.

[12] Sico JJ, Chang CC, So-Armah K, Justice AC, Hylek E, Skanderson M, McGinnis K, Kuller LH, Kraemer KL, Rimland D (2015). HIV status and the risk of ischemic stroke among men. Neurology.

[13] Minciullo PL, Catalano A, Mandraffino G, Casciaro M, Crucitti A, Maltese G, Morabito N, Lasco A, Gangemi S, Basile G (2016). Inflammaging and anti-inflammaging: the role of cytokines in extreme longevity. Arch Immunol Ther Exp (Warsz).

[14] Nasi M, De Biasi S, Gibellini L, Bianchini E, Pecorini S, Bacca V, Guaraldi G, Mussini C, Pinti M, Cossarizza A (2017). Ageing and inflammation in patients with HIV infection. Clin Exp Immunol.

[15] Benjamin LA, Bryer A, Emsley HC, Khoo S, Solomon T, Connor MD (2012). HIV infection and stroke: current perspectives and future directions. Lancet Neurol.

[16] Benjamin LA, Bryer A, Lucas S, Stanley A, Allain TJ, Joekes E, Emsley H, Turnbull I, Downey C, Toh CH (2016). Arterial ischemic stroke in HIV: defining and classifying etiology for research studies. Neurol Neuroimmunol Neuroinflamm.

[17] Hammond CK, Eley B, Wieselthaler N, Ndondo A, Wilmshurst JM (2016). Cerebrovascular disease in children with HIV-1 infection. Dev Med Child Neurol.

[18] Pathai S, Bajillan H, Landay AL, High KP (2014). Is HIV a model of accelerated or accentuated aging?. J Gerontol A Biol Sci Med Sci.

[19] Raghavan A, Rimmelin DE, Fitch KV, Zanni MV (2017). Sex differences in select non-communicable HIV-associated comorbidities: exploring the role of systemic immune activation/inflammation. Curr HIV/AIDS Rep.

[20] Forbes HJ, Williamson E, Benjamin L, Breuer J, Brown MM, Langan SM, Minassian C, Smeeth L, Thomas SL, Warren-Gash C (2018). Association of herpesviruses and stroke: systematic review and meta-analysis. PLoS ONE.

[21] Zhang Y, Luo G, Huang Y, Yu Q, Wang L, Li K (2017). Risk of stroke/transient ischemic attack or myocardial infarction with herpes zoster: A systematic review and meta-analysis. J Stroke Cerebrovasc Dis.

[22] Hatleberg CI, Ryom L, Kamara D, De Wit S, Law M, Phillips A, Reiss P, D'Arminio Monforte A, Mocroft A, Pradier C (2019). Predictors of ischemic and hemorrhagic strokes among people living with HIV: the D:A: D International prospective multicohort study. EClinicalmedicine.

[23] Silva-Pinto A, Costa A, Serrão R, Sarmento A, Abreu P (2017). Ischaemic stroke in HIV-infected patients: A case-control study. HIV Med.

[24] Kang JH, Ho JD, Chen YH, Lin HC (2009). Increased risk of stroke after a herpes zoster attack: a population-based follow-up study. Stroke.

[25] Langan SM, Minassian C, Smeeth L, Thomas SL (2014). Risk of stroke following herpes zoster: a self-controlled case-series study. Clin Infect Dis.

[26] Asiki G, Stockdale L, Kasamba I, Vudriko T, Tumwekwase G, Johnston T, Kaleebu P, Kamali A, Seeley J, Newton R (2015). Pilot study of antibodies against varicella zoster virus and human immunodeficiency virus in relation to the risk of developing stroke, nested within a rural cohort in Uganda. Trop Med Int Health.

[27] Patil A, Goldust M, Wollina U (2022). Herpes zoster: A review of clinical manifestations and management. Viruses.

[28] Re H-S (1965). The nature of herpes zoster: A long-term study and a new hypothesis. Proc R Soc Med.

[29] Patterson BJ, Rausch DA, Irwin DE, Liang M, Yan S, Yawn BP (2019). Analysis of vascular event risk after herpes zoster from 2007 to 2014 US insurance claims data. Mayo Clin Proc.

[30] Lin LY, Warren-Gash C, Smeeth L, Chen PC (2018). Data resource profile: the National Health Insurance Research Database (NHIRD). Epidemiol Health.

[31] Hsieh CY, Su CC, Shao SC, Sung SF, Lin SJ, Kao Yang YH, Lai EC (2019). Taiwan’s National Health Insurance Research Database: past and future. Clin Epidemiol.

[32] Hsieh MT, Huang KC, Hsieh CY, Tsai TT, Chen LC, Sung SF (2021). Validation of ICD-10-CM diagnosis codes for identification of patients with acute hemorrhagic stroke in a National Health Insurance Claims Database. Clin Epidemiol.

[33] Hsieh MT, Hsieh CY, Tsai TT, Wang YC, Sung SF (2020). Performance of ICD-10-CM diagnosis codes for identifying acute ischemic stroke in a National Health Insurance Claims Database. Clin Epidemiol.

[34] Liu CY, Hung YT, Chuang YL, Chen YJ, Weng WS, Liu JS, Liang KY (2006). Incorporating development stratification of Taiwan townships into sampling design of large scale health interview survey. J Health Manag.

[35] Vinikoor MJ, Napravnik S, Floris-Moore M, Wilson S, Huang DY, Eron JJ (2013). Incidence and clinical features of cerebrovascular disease among HIV-infected adults in the Southeastern United States. AIDS Res Hum Retroviruses.

[36] Chow FC, Wilson MR, Wu K, Ellis RJ, Bosch RJ, Linas BP (2018). Stroke incidence is highest in women and non-Hispanic blacks living with HIV in the AIDS Clinical Trials Group Longitudinal Linked Randomized Trials cohort. AIDS.

[37] Gutierrez J, Hatleberg CI, Evans H, Yin MT (2019). Role of pre-stroke immunity in ischemic stroke mechanism among patients with HIV. AIDS Care.

[38] Sundström K, Weibull CE, Söderberg-Löfdal K, Bergström T, Sparén P, Arnheim-Dahlström L (2015). Incidence of herpes zoster and associated events including stroke—a population-based cohort study. BMC Infect Dis.

[39] Juma K, Nyabera R, Mbugua S, Odinya G, Jowi J, Ngunga M, Zakus D, Yonga G (2019). Cardiovascular risk factors among people living with HIV in rural Kenya: A clinic-based study. Cardiovasc J Afr.

[40] Wu PH, Chuang YS, Lin YT (2019). Does herpes zoster increase the risk of stroke and myocardial infarction? A comprehensive review. J Clin Med.

[41] Nagel MA, Traktinskiy I, Azarkh Y, Kleinschmidt-DeMasters B, Hedley-Whyte T, Russman A, VanEgmond EM, Stenmark K, Frid M, Mahalingam R (2011). Varicella zoster virus vasculopathy: analysis of virus-infected arteries. Neurology.

[42] Benjamin LA, Allain TJ, Mzinganjira H, Connor MD, Smith C, Lucas S, Joekes E, Kampondeni S, Chetcuti K, Turnbull I (2017). The role of human immunodeficiency virus-associated vasculopathy in the etiology of stroke. J Infect Dis.

[43] Erskine N, Tran H, Levin L, Ulbricht C, Fingeroth J, Kiefe C, Goldberg RJ, Singh S (2017). A systematic review and meta-analysis on herpes zoster and the risk of cardiac and cerebrovascular events. PLoS ONE.

[44] Lian Y, Zhu Y, Tang F, Yang B, Duan R (2017). Herpes zoster and the risk of ischemic and hemorrhagic stroke: A systematic review and meta-analysis. PLoS ONE.

[45] Minassian C, Thomas SL, Smeeth L, Douglas I, Brauer R, Langan SM (2015). Acute cardiovascular events after herpes zoster: A self-controlled case series analysis in vaccinated and unvaccinated older residents of the United States. PLoS Med.

