# Supplementary material for: Herpes zoster associated with stroke incidence in people living with human immunodeficiency virus: a nested case–control study
Source: BMC Infect Dis. 2023 Sep 28;23:636. doi: 10.1186/s12879-023-08628-8 (PMC10536781; doi:10.1186/s12879-023-08628-8)

**Herpes Zoster Associated with Stroke Incidence in People Living with Human Immunodeficiency Virus: A Nested Case-Control Study**

Supplementary Table 1: Operation definitions for study outcomes

| **Outcome** | **ICD-9-CM disease code** | **ICD-10-CM disease code** |
| --- | --- | --- |
| Comorbidities | | |
| Hypertension | 401-405 | I10-I13, I15 |
| Diabetes mellitus | 250 | E11.9 |
| Hyperlipidemia | 272.0-272.4 | E10-E14 |
| Coronary artery disease | 410.x, 411.x, 412.x, 413.x, 414.x | I20, I21-I22, I24, I25 |
| Acute myocardial infarction | 410-410.9, 412 | I21-I22, I25.2 |
| Heart failure | 428 | I50.x |
| Atrial fibrillation | 427.31 | I48.0, I48.2, I48.91 |
| Chronic kidney disease | 585.9 | N18.4, N18.5, N18.6, N18.9 |
| Hepatitis B virus | 070.2, 070.3 | B16.0, B16.1, B16.2, B16.9, B18.0, B18.1, B19.10, B19.11 |
| Hepatitis C virus | 070.4, 070.41, 070.44, 070.51, 070.54, V02.62 | B17.0, B17.10, B17.11, B17.2, B17.8, B18.2, B19.20, B19.21, Z22.52 |

Supplementary Figure 1: Overview of the participant selection process


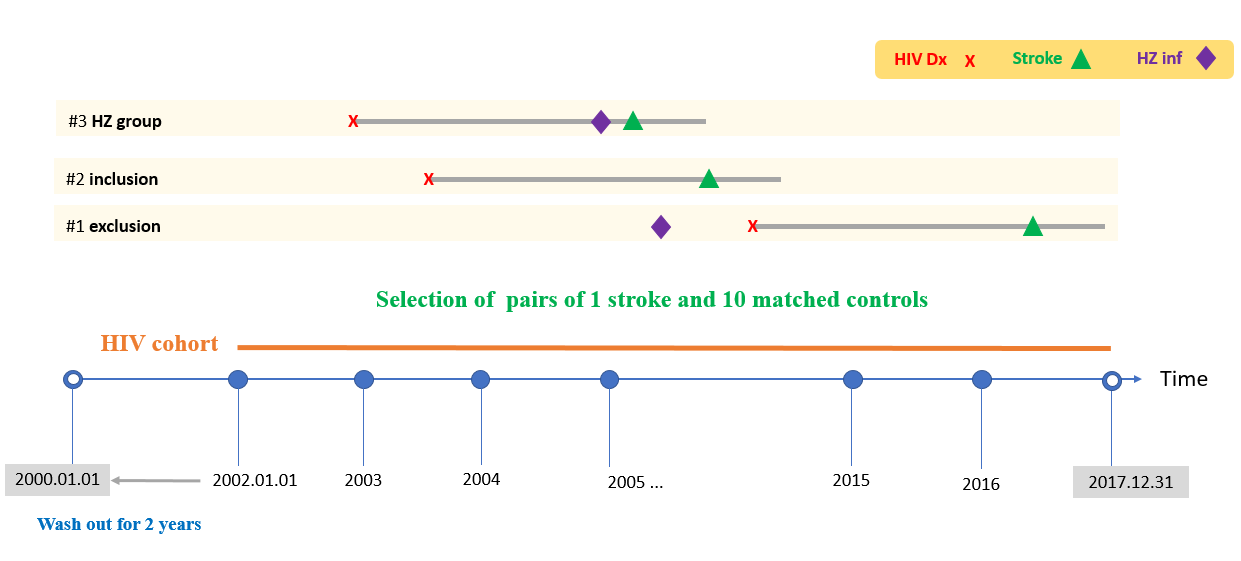

Supplement: Supplementary file 1 — Additional file 1: Supplementary Table 1. Operation definitions for study outcomes. Supplementary Figure 1. Overview of the participant selection process. [file 12879_2023_8628_MOESM1_ESM.docx]
